# Type 2 diabetes pathway-specific polygenic risk scores elucidate heterogeneity in clinical presentation, disease progression and diabetic complications in 18,217 Chinese individuals with type 2 diabetes

**DOI:** 10.1007/s00125-024-06309-y

**Published:** 2024-11-12

**Authors:** Gechang Yu, Claudia H. T. Tam, Cadmon K. P. Lim, Mai Shi, Eric S. H. Lau, Risa Ozaki, Heung-man Lee, Alex C. W. Ng, Yong Hou, Baoqi Fan, Chuiguo Huang, Hongjiang Wu, Aimin Yang, Hoi Man Cheung, Ka Fai Lee, Shing Chung Siu, Grace Hui, Chiu Chi Tsang, Kam Piu Lau, Jenny Y. Y. Leung, Elaine Y. N. Cheung, Man Wo Tsang, Grace Kam, Ip Tim Lau, June K. Y. Li, Vincent T. F. Yeung, Emmy Lau, Stanley Lo, Samuel Fung, Yuk Lun Cheng, Cheuk Chun Szeto, The Hong Kong Diabetes Biobank Study Group, Elaine Chow, Alice P. S. Kong, Wing Hung Tam, Andrea O. Y. Luk, Michael N. Weedon, Wing-yee So, Juliana C. N. Chan, Richard A. Oram, Ronald C. W. Ma, for the TRANSCEND Consortium

**Affiliations:** 1 Department of Medicine and Therapeutics, The Chinese University of Hong Kong, Hong Kong, China; 2 Hong Kong Institute of Diabetes and Obesity, The Chinese University of Hong Kong, Hong Kong, China; 3 CUHK-SJTU Joint Research Centre in Diabetes Genomics and Precision Medicine, The Chinese University of Hong Kong, Hong Kong, China; 4 Laboratory for Molecular Epidemiology in Diabetes, Li Ka Shing Institute of Health Sciences, The Chinese University of Hong Kong, Hong Kong, China; 5 Diabetes Research Laboratory, Li Ka Shing Institute of Health Sciences, The Chinese University of Hong Kong, Hong Kong, China; 6 Department of Medicine and Geriatrics, Kwong Wah Hospital, Hong Kong, China; 7 Diabetes Centre, Tung Wah Eastern Hospital, Hong Kong, China; 8 Diabetes and Education Centre, Alice Ho Miu Ling Nethersole Hospital, Hong Kong, China; 9 North District Hospital, Hong Kong, China; 10 Department of Medicine and Geriatrics, Ruttonjee Hospital, Hong Kong, China; 11 Department of Medicine and Geriatrics, United Christian Hospital, Hong Kong, China; 12 Tseung Kwan O Hospital, Hong Kong, China; 13 Department of Medicine, Yan Chai Hospital, Hong Kong, China; 14 Centre for Diabetes Education and Management, Our Lady of Maryknoll Hospital, Hong Kong, China; 15 Department of Medicine, Pamela Youde Nethersole Eastern Hospital, Hong Kong, China; 16 Department of Medicine and Geriatrics, Princess Margaret Hospital, Hong Kong, China; 17 Department of Medicine, Alice Ho Miu Ling Nethersole Hospital, Hong Kong, China; 18 Li Ka Shing Institute of Health Sciences, The Chinese University of Hong Kong, Hong Kong, China; 19 Department of Obstetrics and Gynaecology, The Chinese University of Hong Kong, Hong Kong, China; 20 CUHK Medical Centre, Hong Kong, China; 21 University of Exeter Medical School, Exeter, UK

**Keywords:** Chinese population, Diabetic complications, Disease progression, Heterogeneity, Pathway-specific polygenic risk score, Type 2 diabetes

## Abstract

**Aims/hypothesis:**

Type 2 diabetes is a complex and heterogeneous disease and the aetiological components underlying the heterogeneity remain unclear in the Chinese and East Asian population. Therefore, we aimed to investigate whether specific pathophysiological pathways drive the clinical heterogeneity in type 2 diabetes.

**Methods:**

We employed newly developed type 2 diabetes hard-clustering and soft-clustering pathway-specific polygenic risk scores (psPRSs) to characterise individual genetic susceptibility to pathophysiological pathways implicated in type 2 diabetes in 18,217 Chinese patients from Hong Kong. The ‘total’ type 2 diabetes polygenic risk score (PRS) was summed by genome-wide significant type 2 diabetes signals (*n*=1289). We examined the associations between psPRSs and cardiometabolic profile, age of onset, two glycaemic deterioration outcomes (clinical requirement of insulin treatment, defined by two consecutive HbA_1c_ values ≥69 mmol/mol [8.5%] more than 3 months apart during treatment with two or more oral glucose-lowering drugs, and insulin initiation), three renal (albuminuria, end-stage renal disease and chronic kidney disease) outcomes and five cardiovascular outcomes.

**Results:**

Although most psPRSs and total type 2 diabetes PRS were associated with an earlier and younger onset of type 2 diabetes, the psPRSs showed distinct associations with clinical outcomes. In particular, individuals with normal weight showed higher psPRSs for beta cell dysfunction and lipodystrophy than those who were overweight. The psPRSs for obesity were associated with faster progression to clinical requirement of insulin treatment (adjusted HR [95% CI] 1.09 [1.05, 1.13], *p*<0.0001), end-stage renal disease (1.10 [1.04, 1.16], *p*=0.0007) and CVD (1.10 [1.05, 1.16], *p*<0.0001) while the psPRSs for beta cell dysfunction were associated with reduced incident end-stage renal disease (0.90 [0.85, 0.95], *p*=0.0001) and heart failure (0.83 [0.73, 0.93], *p*=0.0011). Major findings remained significant after adjusting for a set of clinical variables.

**Conclusions/interpretation:**

Beta cell dysfunction and lipodystrophy could be the driving pathological pathways in type 2 diabetes in individuals with normal weight. Genetic risks of beta cell dysfunction and obesity represent two major genetic drivers of type 2 diabetes heterogeneity in disease progression and diabetic complications, which are shared across ancestry groups. Type 2 diabetes psPRSs may help inform patient stratification according to aetiology and guide precision diabetes care.

**Graphical Abstract:**

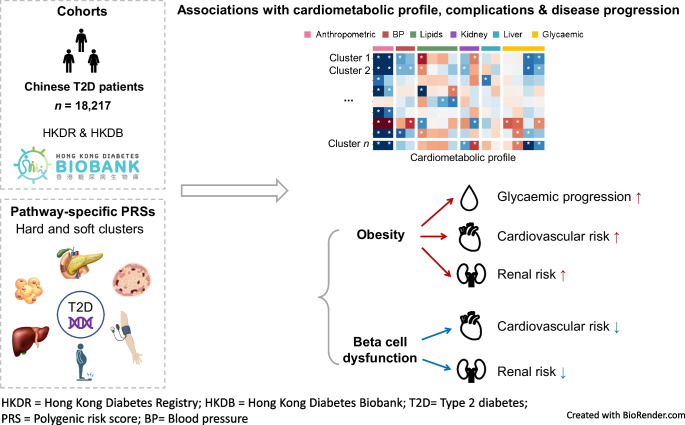

**Supplementary Information:**

The online version of this article (10.1007/s00125-024-06309-y) contains peer-reviewed but unedited supplementary material.


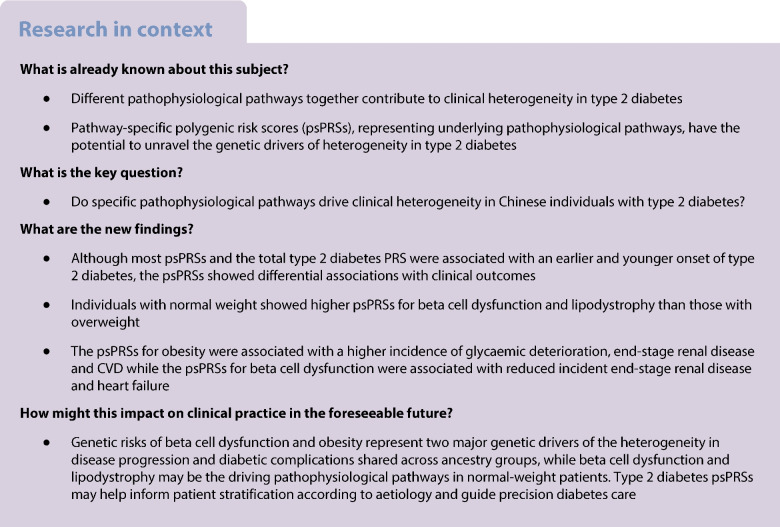


## Introduction

Type 2 diabetes is a complex and heterogeneous disease where aetiology, clinical presentation and prognosis can vary from person to person. There has been increasing awareness that addressing the diabetes heterogeneity plays a pivotal role in precision treatment and management [1–3]. Compared with clinical features, individual genetic information remains mostly unchanged but has profound and lasting impact on disease risk during the lifetime; this could help us better understand the aetiology of heterogeneity [4]. To this end, the partitioned or pathway-specific polygenic risk score (psPRS) for type 2 diabetes, which evaluates genetic predisposition to the specific pathophysiological pathway, has been proposed for characterising and understanding the aetiology of clinical heterogeneity through different pathophysiological pathways [5–9].

Udler and colleagues [5, 7] implemented a ‘soft-clustering’ approach (i.e. a single SNP could belong to multiple clusters), identifying several type 2 diabetes genetic clusters in the European population. Their recent work [8] further extended to 12 multi-ancestry clusters. In parallel, the T2D Global Genomics Initiative [9] performed the largest (*n*>2.5 million) multi-ancestry type 2 diabetes genome-wide association study (GWAS) to date and developed eight type 2 diabetes genetic clusters using a ‘hard-clustering’ approach (i.e. each SNP only belongs to one cluster). However, Chinese and East Asian populations may have different driving pathways compared with the European population [10]. It remains unclear whether these newly developed type 2 diabetes psPRSs could be transferred and applied in Chinese population to find out their distinct driving pathophysiological pathways.

This study utilised the newly developed ‘hard-clustering’ [9] and ‘soft-clustering’ [8] type 2 diabetes psPRSs in 18,217 Chinese individuals with type 2 diabetes from two prospective cohorts in Hong Kong (Hong Kong Diabetes Register [HKDR] and Hong Kong Diabetes Biobank [HKDB]) to investigate whether specific pathophysiological pathways drive the heterogeneity in clinical presentation, disease progression and incident diabetic complications.

## Methods

### Participants

The 18,217 Chinese participants with type 2 diabetes with genotype data were from two prospective cohorts, HKDR and HKDB. HKDR was established in 1995 as a hospital-based research-driven quality improvement programme at the Prince of Wales Hospital [11] and to date has recruited over 10,000 participants with diabetes. HKDB [12] was established in 2014 to study the risk factors and identify multi-omics biomarkers related to diabetes, diabetes complications or other outcomes, and to date has recruited over 22,000 participants. DNA samples of 6550 and 5644 participants with type 2 diabetes from the HKDB were respectively genotyped in two phases: phase 1 (HKDB-P1); and phase 2 (HKDB-P2). Medication use data were collected from the electronic health record linkage from the Hong Kong Health Authority. For baseline demographics and clinical characteristics of HKDR, HKDB-P1 and HKDB-P2 participants, please see Table 1. For further details, please refer to electronic supplementary material (ESM) Methods (Cohort descriptions).

**Table 1:** Baseline characteristics of the cohorts of Chinese participants with type 2 diabetes

Characteristic	HKDR	HKDB-P1	HKDB-P2
*N* after QC	6023	6550	5644
Age, years	57.4±13.3	61.4±11.3	60.3±12.4
Sex, male	2727 (45.3)	2693 (41.1)	2288 (40.5)
Age at diabetes diagnosis, years	50±12.7	49.7±11.7	49.1±13.0
Duration of diabetes, years	6 (2–11)	11 (4–17)	10 (4–17)
Young-onset diabetes	1320 (22.0)	1182 (18.1)	1103 (19.7)
BMI, kg/m^2^	25.2±4.0	26.3±4.5	26.6±4.7
HbA_1c_, mmol/mol	61±19	60±15	61±16
HbA_1c_, %	7.7±1.8	7.6±1.4	7.7±1.5
Triglyceride, mmol/l	1.4 (0.97–2.09)	1.32 (0.94–1.93)	1.38 (0.97–2.00)
HDLC, mmol/l	1.31±0.37	1.24±0.35	1.21±0.34
LDLC, mmol/l	3.11±1.01	2.40±0.78	2.33±0.78
Systolic BP, mmHg	135.4±20.7	135.3±18.0	134.2±17.9
Diastolic BP, mmHg	75.5±10.9	74.8±11.2	74.5±11.4
Urinary ACR, mg/mmol	2.2 (0.8–12.3)	2.9 (1.0–13.6)	2.7 (1.0–12.2)
eGFR, ml/min per 1.73 m^2^	80.2 (63.9–99.6)	77.8 (62.6–92.2)	78.5 (64.5–96.7)
Genotyping platform	Omni2.5 + exome array	Global Screening Array (GSA)	Asian Screening Array (ASA)
Follow-up period, years	17.7 (11.8–23.2)	6.9 (6.6–7.4)	5.6 (5.2–6.0)
Lipid-lowering drugs	1074 (17.9)	4533 (69.8)	3823 (68.3)
Antihypertensive drugs	2762 (45.9)	4881 (75.1)	3972 (70.9)
Oral glucose-lowering drugs	3986 (66.3)	5573 (93.6)	5002 (89.8)
Insulin	560 (9.3)	1961 (30.0)	1946 (34.5)

Data are expressed as mean ± SD, *n* (%) or median (IQR)

### Clinical and laboratory measurements

In the HKDR and HKDB, participants underwent structured assessments at baseline by trained nurses, including demographics, anthropometrics, medical history, clinical examinations and laboratory investigations. Participant’s sex or gender is self-reported. Blood was sampled, after participants had fasted overnight for 8 h, to measure fasting plasma glucose, HbA_1c_, serum creatinine, alanine aminotransferase (ALT) and bilirubin (only in HKDR), and lipid profile (total cholesterol, HDL-cholesterol [HDLC], triglycerides [TG] and LDL-cholesterol [LDLC]). A random spot urinary sample was used to assess albumin/creatinine ratio (ACR). The Chronic Kidney Disease Epidemiology Collaboration (CKD-EPI) equation was used to determine the eGFR [13]. In the HKDR, fasting C-peptide was additionally measured in archived samples to evaluate beta cell function and insulin resistance, which were assessed by HOMA2-B and HOMA2-IR [14], respectively. The two indices were calculated by the web-based HOMA2 calculator based on the updated HOMA2 model [15].

### Clinical outcomes

First, considering that the delay in treatment intensification with insulin (‘therapeutic inertia’ or ‘clinical inertia’) is a prevalent issue in diabetes care and clinical practice [16–18], we used the progression to ‘requirement’ of insulin treatment (insulin initiation or clinical requirement of insulin treatment) to more accurately characterise glycaemic or diabetes progression, consistent with the definition used in DIRECT study [19] and our previous study [12]. Clinical requirement of insulin treatment was defined as two consecutive HbA_1c_ values of ≥69 mmol/mol (8.5%) more than 3 months apart during treatment with two or more oral glucose-lowering drugs (*n*=2919). Insulin initiation was defined as the first use of insulin with ≥180 days of treatment consecutively (*n*=3800). Second, we used three renal outcomes, including incident albuminuria (*n*=3318), chronic kidney disease (CKD, *n*=3041) and end-stage renal disease (ESRD, *n*=1332). Third, for cardiovascular outcomes, we used incident CHD (*n*=1155), ischaemic stroke (*n*=535), heart failure (*n*=893), atrial fibrillation (*n*=730), and a composite endpoint of CVD (*n*=1821) including CHD, stroke and peripheral vascular disease. The complications or outcomes were defined according to ICD-9 codes (http://www.icd9data.com/2007/Volume1/default.htm). For further details, please see ESM Methods (Definitions of diabetes complications). The follow-up time was defined as the period from the baseline visit to the date of the first clinical outcome or endpoint, death or the censor date, whichever came first. The median (IQR) follow-up period in the HKDR is 17.7 years (11.8, 23.2) while those in the HKDB-P1 and HKDB-P2 are 6.9 years (6.6, 7.4) and 5.6 years (5.2, 6.0), respectively. The censor date for the disease progression outcomes was 30 June 2022, while that for other outcomes was 31 December 2019. For the number of cases and controls at baseline and progressors during the follow-up period, please see ESM Table 1.

### Genotyping, quality control and imputation

DNA samples were genotyped using the following arrays: (1) Illumina Omni2.5 + Exome Array for HKDR; (2) Infinium Global Screening Array for HKDB-P1; and (3) Infinium Asian Screening Array for HKDB-P2. We also tested the utility of additional genotyping by Exome Array in HKDR in PRS calculation compared with HKDB. Please see ESM Methods (Genotyping, quality control and imputation) and ESM Tables 2 and 3 for details. We applied the same standard quality control (QC) procedures on the genotype data in each dataset. For SNP-level QC, we only included biallelic autosomal SNPs and excluded SNPs with the following characteristics: (1) Hardy–Weinberg equilibrium *p*<1×10^−4^; (2) minor allele frequency (MAF) <1%; (3) call rate <95%; or (4) MAF ≥1% but ≤5% if the call rate was <99%. Individual-level QC included the following procedures: (1) sex checking based on the genotype call from chromosome X; (2) samples’ quality checking based on call rate and heterozygosity rate; (3) detecting possible familial relationship using identity-by-descent; and (4) controlling population stratification by performing principal component (PC) analysis on a set of autosomal LD (linkage disequilibrium)-pruned SNPs (*r*^2^<0.2) with MAF ≥1% after excluding the MHC and regions of high LD. The number of participants included in each cohort after QC is listed in Table 1. In each cohort, we imputed the genotype data after QC with the 1000 Genomes Project phase III reference panel and the Michigan Imputation Server using minimac [20]. Imputed SNPs with low imputation quality (*r*^2^<0.5) were excluded from the subsequent analyses.

### psPRSs

We used two newly developed multi-ancestry hard-clustering [9] and soft-clustering [8] type 2 diabetes genetic clusters to calculate psPRSs in our Chinese population, including eight hard-clustering clusters and 12 soft-clustering clusters. Each cluster was defined according to its genetic associations with cardiometabolic traits, and represented a distinct pathophysiological pathway implicated in type 2 diabetes. For example, there were two clusters related to beta cell dysfunction in both approaches: ‘Beta cell + proinsulin (PI)’ and ‘Beta cell−PI’ from hard-clustering; and ‘Beta Cell 1’ and ‘Beta Cell 2’ from soft-clustering. To be specific, in hard-clustering, the Beta cell+PI cluster was positively correlated with proinsulin while the Beta cell−PI cluster showed negative correlation. In soft-clustering, the Beta Cell 1 cluster is more closely related to glucose homeostasis while the Beta Cell 2 cluster is more related to insulin processing. Furthermore, there is a cluster named ‘Obesity’ in both hard- and soft-clustering. For a more detailed description of each cluster, see ESM Tables 4 and 5. The psPRS for each cluster was calculated as the sum of doses of risk alleles weighted by East Asian-specific effect size from the original type 2 diabetes GWAS [9] (hard-clustering) or clustering weights from soft-clustering [8] using Plink v2.0 [21] (https://www.cog-genomics.org/plink/2.0/). ‘Total’ type 2 diabetes PRS was summed by genome-wide independent SNPs (*n*=1289) in Suzuki et al [9] weighted by East Asian-specific effect size. For missing SNPs in each cohort, we searched the LDproxy in LDlink [19] to find proxy SNPs (LD *R*^2^>0.8) in the East Asian population for each missing SNP. We used the *z* score of transformed psPRS (per SD) in each cohort for subsequent statistical analyses. For the allele frequency, effect size and proxy SNPs of each cluster in our study, see ESM Tables 6 and 7.

### Statistical analysis

We first examined the pairwise correlations between hard- and soft-clustering clusters in 18,217 Chinese individuals with type 2 diabetes. Next, the associations with age at diagnosis of type 2 diabetes and young-onset diabetes (YOD, <40 years) were examined. For clinical presentation, we examined the associations with baseline cardiometabolic profile (anthropometric, BP, lipid, kidney, liver and glycaemic measures) using linear regression. Age, sex, duration of type 2 diabetes and the top four PCs were adjusted. In addition, we compared the psPRSs between overweight (reference, BMI >23 kg/m^2^), normal-weight (18.5–23 kg/m^2^) and underweight (BMI <18.5 kg/m^2^) subgroups. For disease progression and incident diabetic complications, we examined the prospective associations with the following variables by Cox regression model adjusted for age, sex, duration of type 2 diabetes and top 4 PCs: (1) clinical requirement of insulin treatment and insulin initiation; (2) albuminuria, CKD and ESRD; and (3) CHD, ischaemic stroke, heart failure, atrial fibrillation and CVD. Participants with a history of the outcome at baseline were excluded from Cox regression. We meta-analysed all the results of clinical variables and outcomes from the three datasets using R package meta [22]. Heterogeneity among datasets was assessed by Cochran’s *Q* statistic [23, 24], Higgins & Thompson’s *I*^2^ statistic [25] and *H*^2^ statistic [25]. We used the Bonferroni method to correct for multiple comparisons. The statistical significance threshold is *p*<0.0056 (0.05/9, 8 clusters + 1 total PRS) for hard-clustering clusters and *p*<0.0042 (0.05/12, 12 clusters) for soft-clustering clusters. In sensitivity analyses, we adopted a more stringent threshold to further correct for the number of clinical outcomes (*n*=10): *p*<0.0006 for hard-clustering clusters and *p*<0.0004 for soft-clustering clusters. To assess whether the effects of psPRSs on clinical outcomes were confounded by clinical variables, we additionally adjusted for the following variables: (1) BMI; and (2) BMI, HbA_1c_, LDLC, TG and systolic BP in the models.

## Results

### Correlations between hard- and soft-clustering clusters

Most correlations between hard- and soft-clustering psPRSs in the Chinese participants with type 2 diabetes were very weak (ESM Fig. 1, ESM Table 8), suggesting that the two clustering approaches are likely to be orthogonal between different clusters. The strongest correlation was observed between the Liver/lipid metabolism cluster from hard-clustering and the Cholesterol cluster from soft-clustering (*r*=0.80). The Obesity clusters from hard- and soft-clustering showed moderate correlation (*r*=0.52). The Beta cell+PI cluster from hard-clustering and Beta Cell 2 cluster from soft-clustering also showed moderate correlation (*r*=0.53).

### Associations with age at diagnosis and risk of young onset

**Fig. 1:**
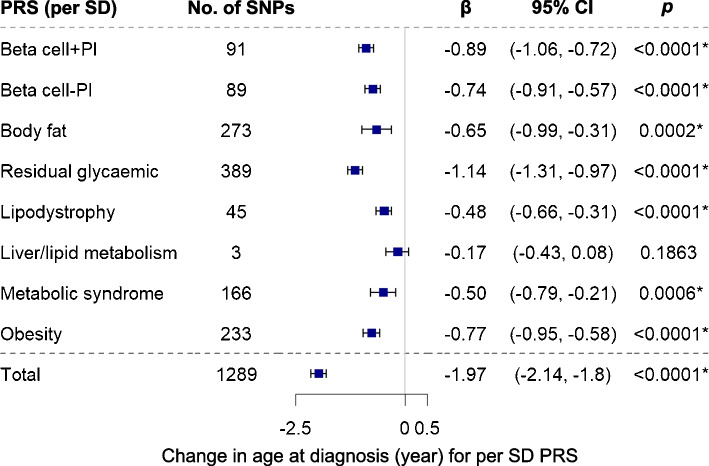
Associations between hard-clustering psPRSs and total type 2 diabetes PRS and age at type 2 diabetes onset in the meta-analysis of over 18,000 Chinese individuals with type 2 diabetes. βs were adjusted for sex and top four PCs. The error bars indicate 95% CI. *p<0.05, Bonferroni corrected

The total type 2 diabetes PRS was strongly associated with an earlier onset (Fig. 1 and ESM Fig. 2, ESM Table 9) and young onset of type 2 diabetes (age <40 years) (ESM Figs 3, 4, ESM Table 10). One SD increase of total type 2 diabetes PRS was related to, on average, a 2 years’ earlier (*p*=1.1×10^−116^) onset among Chinese participants with type 2 diabetes (Fig. 1). Likewise, most psPRSs were associated with an earlier and young onset of type 2 diabetes. In hard-clustering clusters (ESM Tables 9, 10), the psPRSs for Residual glycaemic and Beta cell+PI clusters, respectively, showed the strongest association with earlier onset (β [95% CI] −1.14 [−1.31, −0.97], *p*=3.3×10^−38^) and young onset (OR [95% CI] 1.15 [1.11, 1.20], *p*=5.8×10^−14^) of type 2 diabetes. In soft-clustering clusters, the psPRSs for the Beta Cell 1 cluster showed the strongest association with earlier onset (β [95% CI] −0.66 [−0.83, −0.49], *p*=2.9×10^−14^) and young onset (OR [95% CI] 1.13 [1.09, 1.17], *p*=1.1×10^−10^) of type 2 diabetes.

### Associations with cardiometabolic profile

**Fig. 2:**
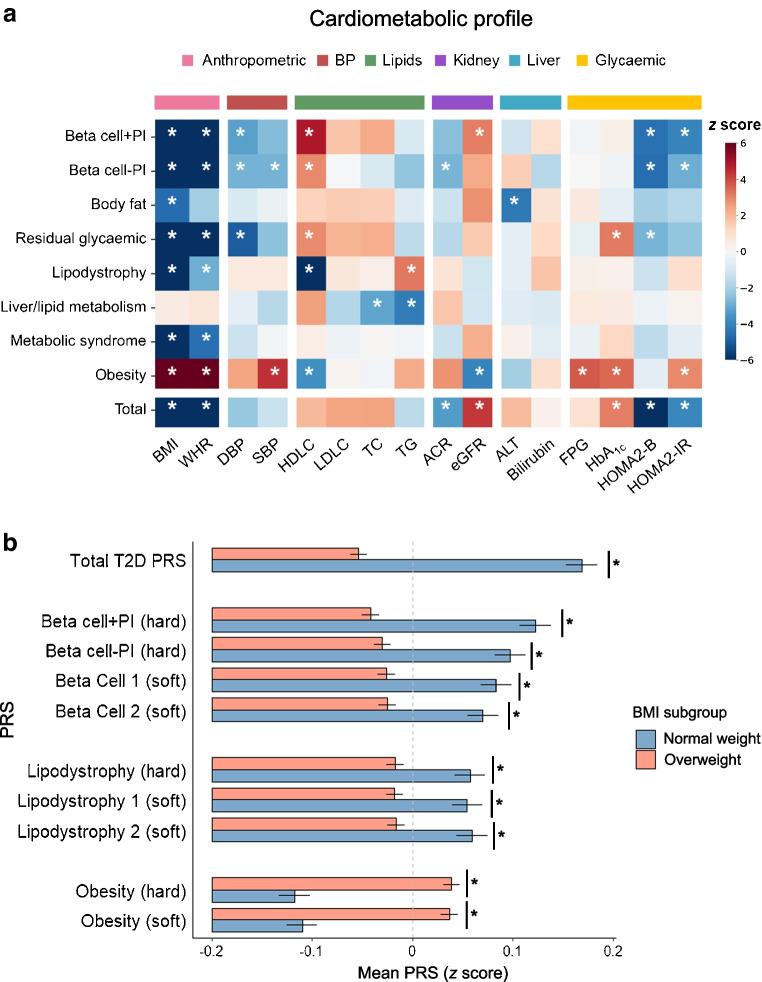
(**a**) Associations of hard-clustering psPRSs and total type 2 diabetes PRS with baseline clinical variables. (**b**) psPRSs and total type 2 diabetes PRS in normal-weight and overweight participants. Age, sex, duration of type 2 diabetes and top four PCs were adjusted in hypothesis testing. The colour bar indicates the *z* score of the regression coefficient. The error bars in (**b**) indicate SD. *p<0.05, Bonferroni corrected. DBP, diastolic BP; FPG, fasting plasma glucose; SBP, systolic blood pressure; T2D, type 2 diabetes

The psPRSs for type 2 diabetes from both hard- and soft-clustering displayed differential associations with cardiometabolic profile at baseline (Fig. 2a and ESM Fig. 5, ESM Table 11), and the association patterns were similar between the two approaches. In particular, most psPRSs and the total type 2 diabetes PRS were associated with lower BMI and WHR while only the psPRS for Obesity was associated with higher BMI and WHR. For insulin deficiency and resistance, the psPRSs for beta cell dysfunction and the total type 2 diabetes PRS were associated with lower HOMA2-B and HOMA2-IR while the psPRS for Obesity was associated with higher HOMA2-IR. Moreover, for the lipid profile, the psPRSs for beta cell dysfunction were associated with higher HDLC and lower TG while the psPRSs for Lipodystrophy and Obesity were associated with lower HDLC and higher TG. Further, compared with overweight participants with type 2 diabetes (*n*=13,650), normal-weight participants (*n*=4134) showed higher total type 2 diabetes PRS and psPRSs for beta cell dysfunction and lipodystrophy but lower psPRSs for Obesity (Fig. 2b and ESM Table 12).

### Prospective associations with disease progression

**Fig. 3:**
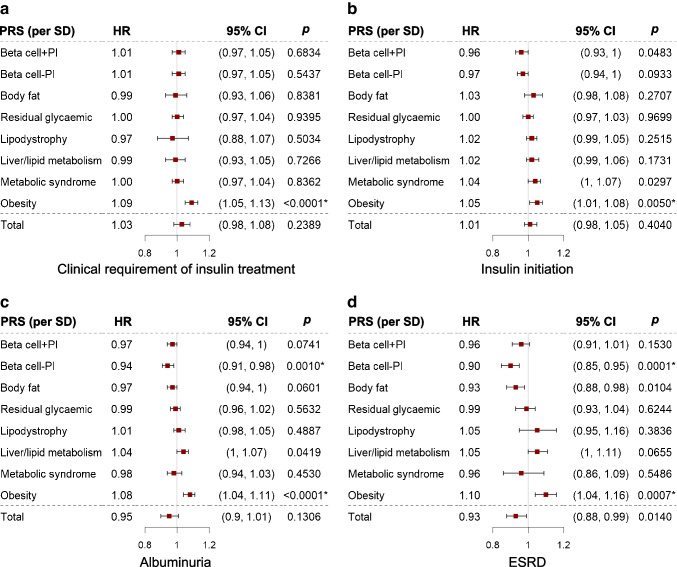
Prospective associations with clinical requirement of insulin treatment (**a**), insulin initiation (**b**), albuminuria (**c**) and ESRD (**d**). Age, sex, duration of type 2 diabetes and top four PCs were adjusted. *p<0.05, Bonferroni corrected. The error bars indicate 95% CI. For the numbers of progressors and non-progressors of each outcome, see ESM Table 1

The psPRS for Obesity from hard-clustering (Fig. 3a, b and ESM Table 13) was significantly associated with a higher incidence of clinical requirement of insulin treatment (HR 1.09 [95% CI 1.05, 1.13], *p*= 9.3×10^−6^) and insulin initiation (HR 1.05 [95% CI 1.01, 1.08], *p*=0.0050). The psPRS for Obesity from soft-clustering (ESM Fig. 6) was also nominally associated with a higher incidence of insulin initiation.

### Prospective associations with renal outcomes

The psPRSs for Beta cell−PI and Obesity from hard-clustering showed opposite effects on renal complications (Fig. 3c, d and ESM Fig. 7, ESM Table 14). The psPRS for Beta cell−PI was significantly associated with a lower incidence of albuminuria (HR 0.94 [95% CI 0.91, 0.98], *p*=0.0010), ESRD (HR 0.90 [95% CI 0.85, 0.95], *p*=0.0001) and CKD (HR 0.94 [95% CI 0.91, 0.98], *p*=0.0016), while the psPRS for Obesity was significantly associated with a higher incidence of albuminuria (HR1.08 [95% CI 1.04, 1.11], *p*=3.1×10^−5^) and ESRD (HR 1.10 [95% CI 1.04, 1.16], *p*=0.0007). Similarly, the psPRS for Obesity from soft-clustering (ESM Fig. 8, ESM Table 14) was also significantly associated with a higher incidence of albuminuria (HR 1.06 [95% CI 1.03, 1.10], *p*=0.0007).

### Prospective associations with incident cardiovascular outcomes

**Fig. 4:**
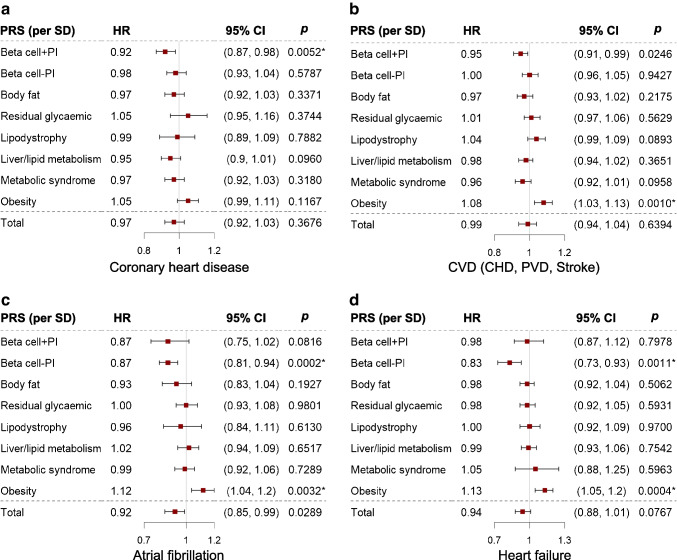
Prospective associations with incident CHD (**a**), CVD (**b**), atrial fibrillation (**c**) and heart failure (**d**) in the HKDR and HKDB. HRs were adjusted for age, sex, duration of type 2 diabetes and top four PCs. *p<0.05, Bonferroni corrected. The error bars indicate 95% CI. For the numbers of progressors and non-progressors of each outcome, see ESM Table 1

The psPRSs for beta cell dysfunction and Obesity from both hard- and soft-clustering showed opposite associations with incident cardiovascular outcomes (Fig. 4a–d and ESM Figs 9, 10, ESM Table 15). We found that participants with higher psPRS for Beta cell−PI from hard-clustering had a lower incidence of atrial fibrillation (HR 0.87 [95% CI 0.81, 0.94], *p*=0.0002) and heart failure (HR 0.83 [95% CI 0.73, 0.93], *p*=0.0011). In addition, participants with a higher psPRS for Beta cell+PI showed a lower incidence of CHD (HR 0.92 [95% CI 0.87, 0.98], *p*=0.0052). Similarly, a higher psPRS for the Beta Cell 2 cluster from soft-clustering was significantly associated with lower incidence of CHD (HR 0.92 [95% CI 0.87, 0.97], *p*=0.0036). On the contrary, participants with higher psPRS for Obesity from hard-clustering had a higher incidence of CVD (HR 1.08 [95% CI 1.03, 1.13], *p*=0.0052), atrial fibrillation (HR 1.12 [95% CI 1.04, 1.20], *p*=0.0032) and heart failure (HR 1.13 [95% CI 1.05, 1.20], *p*=0.0004). Likewise, a higher psPRS for Obesity from soft-clustering was significantly associated with a higher incidence of ischaemic stroke (HR 1.14 [95% CI 1.04, 1.24], *p*=0.0029) and CVD (HR 1.10 [95% CI 1.05, 1.16], *p*=2.3×10^−5^). However, the total type 2 diabetes PRS did not show any significant association with the aforementioned clinical outcomes.

### Sensitivity analysis

After further correcting for the number of examined clinical outcomes using the Bonferroni correction method, some prospective associations survived this more stringent threshold. In the hard-clustering clusters, the psPRS for Beta cell−PI still showed significant associations with lower incidence of ESRD and atrial fibrillation while the psPRS for Obesity showed significant associations with a higher incidence of glycaemic deterioration, albuminuria and heart failure. In the soft-clustering clusters, the Obesity cluster also showed significant association with a higher incidence of CVD. After additionally adjusting for BMI in the models, the significance of the major findings for the psPRSs for beta cell dysfunction and Obesity were only slightly attenuated (ESM Table 16). Most of the significant associations with clinical outcomes still passed the Bonferroni threshold. When further adjusting for BMI, HbA_1c_, LDLC, TG and systolic BP in the models, the major findings remained significant (ESM Table 17). In particular, The psPRSs for Obesity were associated with a higher incidence of clinical requirement of insulin treatment (HR 1.06 [95% CI 1.02, 1.10], *p*=0.0027), albuminuria (HR 1.05 [95% CI 1.02, 1.09], *p*=0.0041) and CVD (HR 1.08 [95% CI 1.03, 1.13], *p*=0.0021) while the psPRS for beta cell dysfunction was associated with a lower incidence of heart failure (HR 0.85 [95% CI 0.76, 0.95], *p*=0.0050).

## Discussion

In 18,217 Chinese participants with type 2 diabetes, we comprehensively investigated the clinical relevance of psPRSs for type 2 diabetes using newly developed hard- and soft-clustering psPRSs. While the total type 2 diabetes PRS and individual psPRSs were consistent in showing associations with an earlier and young onset of type 2 diabetes, the psPRSs had differential associations with cardiometabolic profile and clinical outcomes. In particular, normal-weight individuals were burdened with higher genetic risks of beta cell dysfunction and lipodystrophy than overweight individuals. The psPRSs for Obesity were associated with faster glycaemic deterioration and increased incident cardiorenal complications while the psPRSs for beta cell dysfunction showed associations with reduced incident cardiorenal complications. Our findings offer novel mechanistic insights into the clinical heterogeneity in Chinese individuals with type 2 diabetes and may facilitate aetiologically informed patient stratification and guide precision diabetes care.

Our major findings are consistent with those in the multi-ancestry type 2 diabetes GWAS study from Suzuki et al [9], which also found that the psPRSs for beta cell dysfunction and Obesity showed opposite directions of effects on risk of cardiorenal complications. In particular, utilising 29,827 European individuals with type 2 diabetes from six clinical trials, they highlighted that the psPRS for beta cell dysfunction was associated with a lower incidence of cardiovascular outcomes and heart failure while the psPRS for Obesity was associated with a higher incidence of heart failure. Our study further showed that individuals with a high psPRS for Obesity displayed more rapid progression to glycaemic deterioration. Our study is the first to examine and compare the prospective associations between hard- and soft-clustering psPRSs, and clinical outcomes in East Asian individuals with type 2 diabetes. These results suggest that the genetic risks of beta cell dysfunction and obesity could be two major genetic drivers of the heterogeneity in disease progression and diabetic complications and are shared across ancestry groups.

Further, compared with overweight individuals with type 2 diabetes, those with normal weight exhibited higher total type 2 diabetes genetic burden and higher pathway genetic burden for beta cell dysfunction and lipodystrophy. Smith et al [8] found that the East Asian population has a higher proportion of polygenic risk related to lipodystrophy. Ojima et al [26] also found that the genetic risk of beta cell dysfunction was mainly enriched in low-BMI individuals. Taken together, these findings suggest beta cell dysfunction and lipodystrophy may be the driving pathological pathways in normal-weight individuals with type 2 diabetes and could be potential treatment or intervention targets in this group.

It is interesting to note that higher psPRSs for beta cell dysfunction were associated with lower incidence of cardiorenal complications. According to a new integrative framework [3], diabetes heterogeneity can be perceived as a representation of different pathophysiological pathways or factors, combining to cause the onset of diabetes (when a risk threshold is exceeded) and influencing the severity and prognosis of diabetes. Therefore, in individuals with higher psPRS for beta cell dysfunction, insulin resistance/obesity is likely to make less of a contribution towards the type 2 diabetes pathophysiology. In contrast, individuals with lower psPRS for beta cell dysfunction may experience more cumulative effects from insulin resistance, obesity or other environmental factors such as sedentary habits and unhealthy diet, contributing to the onset of type 2 diabetes. In addition, these cumulative risk factors could further increase their future cardiorenal risks after the diagnosis of type 2 diabetes. In brief, our study informs which type 2 diabetes genetic risk pathways are most strongly associated with disease progression and complications and strengthens the notion that obesity is a critically important risk factor for progression and complications while highlighting that a genetic predisposition to deficiency in insulin secretion commonly has the opposite associations. Further understanding of these underlying associations may help guide interventions to modify these outcomes.

Further, when additionally adjusting for BMI and other clinical variables, our major findings remained significant. This suggests that the identified prospective associations between psPRSs and clinical outcomes were to some extent independent of clinical variables, underscoring the clinical utility of psPRSs in predicting disease progression and cardiorenal risk. In addition, we found that it was the psPRSs but not the total type 2 diabetes PRS that showed significant and distinct associations with disease progression and clinical outcomes. Therefore, compared with traditional single-dimension PRS for type 2 diabetes, the psPRSs dissect the combined type 2 diabetes genetic risks into distinct pathophysiological pathways and capture their contributions to clinical heterogeneity, which would have been obscured in the total type 2 diabetes PRS.

Our results are complementary to diabetes subgroups or endotypes previously identified using clinical variables in European populations [27–30]. The psPRSs for beta cell dysfunction may be implicated in the severe insulin-deficient diabetes (SIDD) subgroup from Ahlqvist et al [27] clusters while the psPRS for Obesity may be implicated in the severe insulin-resistant diabetes (SIRD) subgroup. Their findings also highlighted the higher incidence of cardiorenal complications in the SIRD subgroup. Most previous clustering studies in East Asian populations [31–35] focused on replicating or extending Ahlqvist clusters using the same or more clinical features. Although the clusters identified in East Asian individuals were similar to Ahlqvist clusters, their associations with cardiorenal risk showed different patterns and their sample sizes were small, and they did not reveal the driving pathways underlying the heterogeneity of cardiorenal risk in the East Asian population. Our study utilised the psPRSs in two large-scale East Asian cohorts (*n*~20,000) and showed that the East Asian population and the European population may share similar driving pathophysiological pathways implicated in cardiorenal risks, suggesting that the psPRSs may be incorporated when addressing diabetes heterogeneity.

We acknowledge that there are some limitations in our study. First, compared with clinical variables [29], the effect size of psPRSs in our study was relatively small. Previous studies [36–39] have shown that, compared with clinical variables or models, PRS alone cannot perform better in predicting the risk of diseases, especially those with high polygenicity. Therefore, rather than test the prediction performance of type 2 diabetes psPRSs, the main aim of our study was to understand the genetic aetiology of type 2 diabetes clinical heterogeneity using type 2 diabetes psPRSs. Future work needs to examine the prediction performance of combining psPRSs and clinical variables. Second, our study did not examine the associations between type 2 diabetes psPRSs and established subgroups or endotypes such as Ahlqvist clusters. It is also important to study the links between the clustering efforts based on clinical variables and genetic information in the future, and findings may be combined to characterise the clinical and aetiological heterogeneity in type 2 diabetes. In addition, although sex was adjusted in our analyses, we did not perform subgroup analyses stratified by sex to see if there was any difference in association patterns between male and female participants, which suggests our findings should be interpreted with caution in terms of sex difference. Finally, since the current follow-up period of the HKDB is short and thus the numbers of event cases are relatively small, further analyses with longer follow-up would be helpful.

Nevertheless, this detailed and comprehensive evaluation in our cohorts of Chinese individuals with type 2 diabetes highlighted that the psPRSs for beta cell dysfunction and obesity could be two major genetic drivers of the heterogeneity in disease progression and diabetic complications shared across ancestry groups while beta cell dysfunction and lipodystrophy may be the driving pathological pathways in normal-weight individuals. Our study suggested the potential of psPRSs for type 2 diabetes in characterising aetiological heterogeneity and addressing the clinical heterogeneity among type 2 diabetes patients, which may help aetiologically and genetically informed patient stratification and further guide precision diabetes care.

## Supplementary Information

Below is the link to the electronic supplementary material.

ESM (PDF 1.93 MB)

ESM Tables (XLSX 469 KB)

## Data Availability

The list of SNPs used to calculate hard- and soft-clustering psPRSs can be found in ESM Tables 6 and 7. HKDR and HKDB data are not publicly available but may be made available upon reasonable request to the corresponding author.

## Abbreviations

ACR: Albumin/creatinine ratio
ALT: Alanine aminotransferase
CKD: Chronic kidney disease
ESRD: End-stage renal disease
GWAS: Genome-wide association study
HDLC: HDL-cholesterol
HKDB: Hong Kong Diabetes Biobank
HKDB-P1: Hong Kong Diabetes Biobank-Phase 1
HKDB-P2: Hong Kong Diabetes Biobank-Phase 2
HKDR: Hong Kong Diabetes Register
LD: Linkage disequilibrium
LDLC: LDL-cholesterol
MAF: Minor allele frequency
PC: Principal component
PI: Proinsulin
PRS: Polygenic risk score
psPRS: Pathway-specific polygenic risk score
QC: Quality control
SIRD: Severe insulin-resistant diabetes
TG: Triglycerides
