# Supplementary material for: Type 2 diabetes pathway-specific polygenic risk scores elucidate heterogeneity in clinical presentation, disease progression and diabetic complications in 18,217 Chinese individuals with type 2 diabetes
Source: Diabetologia. 2024 Nov 12;68(3):602–14. doi: 10.1007/s00125-024-06309-y (PMC11832604; doi:10.1007/s00125-024-06309-y)
Supplement: Supplementary file 1 — ESM (PDF 1.93 MB) [file 125_2024_6309_MOESM1_ESM.pdf]

Supplemental Materials

Methods

Cohort descriptions

The Hong Kong Diabetes Register (HKDR) study

The Hong Kong Diabetes Register (HKDR) baseline study ran from 1995 to 2007 and consists of >10,000 patients with diabetes (1). It was established as a quality improvement program and the study of outcomes for Chinese patients with diabetes at the Prince of Wales Hospital in Hong Kong, which serves a population of over 1.2 million. These patients were referred from hospital-based specialty clinics, community clinics, and general practitioners. The Register consecutively enrolled patients with diabetes who were referred to the Diabetes Mellitus and Endocrine Centre for comprehensive assessment of diabetic complications and metabolic control. Type 2 Diabetes (T2D) was diagnosed according to the 1998 World Health Organization (WHO) criteria. Patients with type 1 diabetes, with acute ketotic presentation, or patients with self-reported non-Chinese or unknown nationality, or missing data on the type of diabetes, or those who had continuous requirement of insulin within one year of diagnosis were excluded. In addition to detailed collection of clinical information and comprehensive assessment of diabetes complications at baseline according to the EURODIAB protocol, participants attended regular repeat diabetes complication assessment. Additional information on hospitalisation, new diagnoses, prescription, and biochemical investigations are available and captured for further definition of endpoints

as described in detail in the next section.

The Hong Kong Diabetes Biobank (HKDB)

The Hong Kong Diabetes Biobank (HKDB) was established as a multi-center diabetes registry and biobank for large-scale replication of genetic and epigenetic markers identified in the project since 2014 (http://www.hongkongdiabetesbiobank.org/, 31st December 2019). It recruited subjects through 11 participating diabetes centers and three renal units at major public hospitals across Hong Kong. Subjects with T2D undergoing routine diabetes complication screening assessment were invited for recruitment into the Biobank. Similar methods and criteria were used for the recruitment in HKDB as in HKDR. Patients with type 1 diabetes, with acute ketotic presentation, or patients with self-reported non-Chinese or unknown nationality, or missing data on the type of diabetes, or those who had continuous requirement of insulin within one year of diagnosis were excluded. To date, more than 22,000 participants have been recruited into HKDB and more than 15,000 participants have genotype data.

Definitions of diabetes complications

The definitions of diabetes complications in HKDR and HKDB were based on the International Classification of Diseases, Ninth Revision (ICD-9). The status of albuminuria in participants from HKDR and HKDB was defined by two consecutive ACR $\geq$ 3 mg/mmol with three months apart. End-stage renal disease (ESRD) is determined if one of the following criteria is met: (1) eGFR<15 (acute kidney failure excluded): A. for history of ESRD before baseline, the eGFR at baseline or the latest eGFR before baseline <15; B. for new event, 2 eGFR<15 separated by 90-365 days; (2)

haemodialysis dialysis with diagnosis of CKD or renal failure, (3) peritoneal dialysis, (4) transplant of kidney, (5) complications of transplanted kidney, (6) persons with a condition influencing their health status; organ or tissue replaced by transplant (kidney). According to KDIGO 2024 Clinical Practice Guideline for the Evaluation and Management of Chronic Kidney Disease (2), CKD is defined as (1) ESRD, (2) eGFR<60 but excluding acute kidney failure: A. for history, the eGFR at baseline or the latest eGFR before baseline <60; B. for new event, 2 eGFR<60 separated by 90-365 days. Coronary heart disease (CHD) is defined as: (1) acute myocardial infarction, (2) other acute and subacute forms of ischemic heart disease, (3) old myocardial infarction, and (4) angina pectoris and other forms of chronic ischemic heart disease. Stroke is defined as: (1) subarachnoid hemorrhage, (2) intracerebral hemorrhage, (3) other and unspecified intracranial hemorrhage, (4) occlusion and stenosis of precerebral arteries, (5) occlusion of cerebral arteries, (6) acute, but ill-defined, cerebrovascular disease (7) other and ill-defined cerebrovascular disease, and (8) late effects of cerebrovascular disease. Peripheral vascular disease (PVD) is defined as: (1) diabetes with peripheral circulatory disorders, (2) gangrene, (3) peripheral angiopathy in diseases classified elsewhere, (4) peripheral vascular disease, unspecified, (5) other peripheral vascular shunt or bypass; insertion of non-drug-eluting peripheral vessel stent(s), and (6) amputation of lower limb but discharges with a traumatic amputation diagnosis code (895-897) were excluded. The composite endpoint of Cardiovascular disease (CVD) is defined as a composite end point as CHD, stroke and PVD. Heart failure (HF) is defined as hospitalisation with heart failure.

Genotyping, quality control and imputation

As the reviewer points out, HKDR was additionally genotyped by exome array. The full name of genotype array is Infinium Omni2.5Exome-8 v1.3 (https://emea.illumina.com/library-prep-array-kit-selector/kits-and-arrays/humanomni25exome-8_8-sample.html). The Infinium Omni2.5Exome-8 BeadChip combines >2.5 million tag SNPs from the HapMap and 1000 Genome Project (>2.5% MAF) with >240,000 markers from the Infinium Exome-24 BeadChip. If additionally combining exome array could reduce the missingness of exome SNPs for psPRSs, the ratio of exome SNPs in missing SNPs in HKDR should be lower than that in HKDB-P1 and HKDB-P2. As ESM Table 2 and 3 show below, the reason why some missing SNPs cannot find proxy SNPs in 1000 Genome Project is due to (1) the missing SNP is monoallelic in East Asian population, or (2) there is no SNP in high LD (r^2>0.8) with the missing SNP. We did functional annotation on these missing SNPs using FUMA [17] and dbSNP from NCBI (https://www.ncbi.nlm.nih.gov/snp/) to find out their category (Exome, Intron, Intergenic or others). We found that the ratio of Exome SNPs in missing SNPs in HKDR is similar to that in HKDB-P1 and HKDB-P2 for both hard- and soft-clustering psPRSs. Therefore, it suggests that additionally combining exome array may not bring significant improvement when calculating PRS in our study.

ESM Table 2. Number of missing SNPs without proxy SNPs in hard-clustering psPRSs

	Total	Exome	Intron	Intergenic
HKDR	147	14 (10%)	84 (57%)	37 (25%)
HKDB-P1	164	11 (7%)	97 (59%)	46 (28%)
HKDB-P2	197	14 (7%)	107 (54%)	59 (30%)

ESM Table 3. Number of missing SNPs without proxy SNPs in soft-clustering psPRSs

	Total	Exome	Intron	Intergenic
HKDR	1	0	1 (100%)	0
HKDB-P1	10	0	8 (80%)	0
HKDB-P2	2	0	2 (100%)	0

Additional information

Hong Kong Diabetes Biobank Study Group Members

Ronald C.W. Ma [1,2,3,4], Juliana C.N. Chan [1,2,3,4], Risa Ozaki[1,2], Andrea O. Luk[1,2,3,4], Wing Yee So[1,2], Cadmon King Poo Lim [1,3], Ka Fai Lee[6], Shing Chung Siu[7], Grace Hui [7], Chiu Chi Tsang [8], Kam Piu Lau[9], Jenny Y.Y. Leung [10], Man Wo Tsang[11], Grace Kam[11], Elaine Cheung[11], Ip Tim Lau [12], June Kam-yin Li[13], Vincent TF Yeung [14], Samuel KS Fung[15], Stanley Lo[16], Emmy Lau[16], Yuk Lun Cheng[17], Stephen Kwok-wing Tsui[18], Yu Huang[18], Hui-yao Lan[1,3], Weichuan Yu[5], Brian Tomlinson[1], Si Lok[19], Ting Fung Chan[19], Kevin Yuk-lap Yip[20], Cheuk Chun Szeto[1,3], Xiaodan Fan[20], Nelson LS Tang[3, 21], Xiaoyu Tian[18], Claudia H.T. Tam[1,4], Guozhi Jiang[1,4], Shi Mai[1,2], Baoqi Fan[1,2], Eric S Lau[1], Fei Xie[1], Sen Zhang[5], Pu Yu[5], Meng Wang[20], Heung Man Lee[1], Fangying Xie[1], Alex C.W. Ng[1,4], Grace Cheung[1], Alice PS Kong[1,2], Elaine Y.K. Chow[1,2], Ming Wai Yeung[1], Chun Chung Chow[1], Kitty K.T. Cheung[1], Rebecca Y.M. Wong[1], So Hon Cheong[18], Katie KH Chan[1,2], Chin-san Law[11], Anthea Ka Yuen Lock[12], Ingrid Kwok Ying Tsang[12], Susanna Chi Pun Chan[12], Yin Wah Chan[12], Cherry Chiu[2], Chi Sang Hung[11], Cheuk Wah Ho[11], Ivy Hoi Yee Ng[11], Juliana Mun Chun Fok[5], Kai Man Lee[6], Hoi Sze Candy Leung[14], Ka Wah Lee[13], Hui Ming Chan[13],

Winnie Wat[16], Tracy Lau[16], Rebecca Law[15], Ryan Chan[15], Candice Lau[1], Pearl Tsang[1], Vince Chan[1], Lap Ying Ho[1], Eva Wong[1], Josephine Chan[1], Sau Fung Lam[1], Jessy Pang[1], Yee Mui Lee[1]

[1] Department of Medicine and Therapeutics, The Chinese University of Hong Kong

[2] Hong Kong Institute of Diabetes and Obesity, The Chinese University of Hong Kong, Hong Kong

[3] Li Ka Shing Institute of Health Sciences, The Chinese University of Hong Kong, Hong Kong

[4] Chinese University of Hong Kong-Shanghai Jiao Tong University Joint Research Centre in Diabetes Genomics and Precision Medicine

[5] Department of Electronic and Computer Engineering, Hong Kong University of Science and Technology

[6] Department of Medicine and Geriatrics, Kwong Wah Hospital

[7] Diabetes Centre, Tung Wah Eastern Hospital, Hong Kong

[8] Diabetes and Education Centre, Alice Ho Miu Ling Nethersole Hospital, Hong Kong

[9] North District Hospital, Hong Kong

[10] Department of Integrated Medical Service, Ruttonjee Hospital, Hong Kong

[11] Diabetes Ambulatory Care Centre, Department of Medicine and Geriatrics, United Christian Hospital

[12] Tseung Kwan O Hospital, Hong Kong

[13] Department of Medicine, Yan Chai Hospital, Hong Kong

[14] Centre for Diabetes Education and Management, Our Lady of Maryknoll Hospital, Hong Kong

[15] Department of Medicine and Geriatrics, Princess Margaret Hospital, Hong Kong

[16] Department of Medicine, Pamela Youde Nethersole Eastern Hospital, Hong Kong.

[17] Department of Medicine, Alice Ho Miu Ling Nethersole Hospital, Hong Kong

[18] School of Biomedical Sciences, Faculty of Medicine, The Chinese University of Hong Kong, Hong Kong

[19] School of Life Sciences, The Chinese University of Hong Kong

[20] Department of Computer Science and Engineering, The Chinese University of Hong Kong

[21] Department of Chemical Pathology, The Chinese University of Hong Kong

TRANSCEND Consortium Group members

Ronald C.W. Ma [1,2,3,4], Juliana C.N. Chan [1,2,3,4], Yu Huang[18], Hui Yao, Si Lok[20], Brian Tomlinson[1], Kwok Wing Stephen Tsui [5], Weichuan Yu[6], Ting Fung Chan[7], Xiaodan Fan[8], Andrea On Yan Luk [1,2,3,4], Wing Yee So[1,2], Cheuk Chun Szeto[1,3], Leung Sang Nelson Tang [9], Xiaoyu Tian[10], Yuk Lap[11]

[1] Department of Medicine and Therapeutics, The Chinese University of Hong Kong

[2] Hong Kong Institute of Diabetes and Obesity, The Chinese University of Hong Kong

[3] Li Ka Shing Institute of Health Sciences, The Chinese University of Hong Kong

[4] Chinese University of Hong Kong-Shanghai Jiao Tong University Joint Research Centre in Diabetes Genomics and Precision Medicine

[5] Hong Kong Bioinformatics Centre, The Chinese University of Hong Kong

[6] School of Life Sciences, The Chinese University of Hong Kong

[7] Department of Statistics, The Chinese University of Hong Kong

[8] CUHK-SJTU Joint Research Centre on Diabetes Genomics and Precision Medicine

[9] Department of Chemical Pathology, The Chinese University of Hong Kong

[10] The Centre for Applied Genomics, The Hospital for Sick Children, Toronto, Canada

[11] Department of Computer Science and Engineering, The Chinese University of Hong Kong

Figures

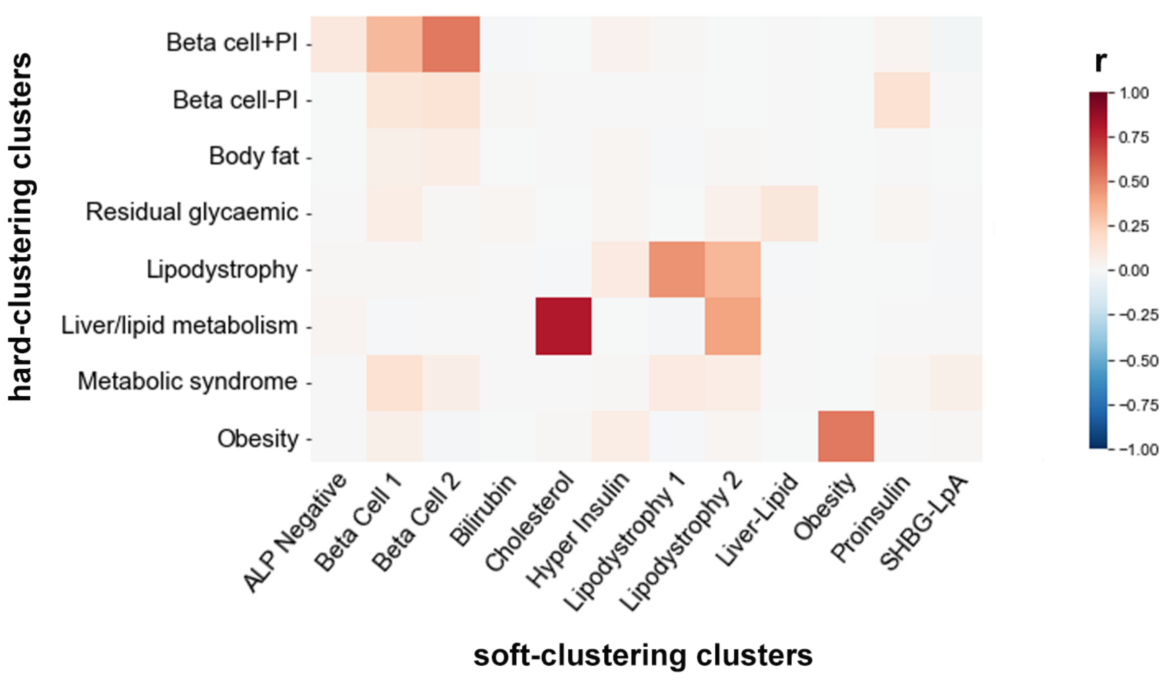

ESM Fig. 1. Pearson correlations between hard-clustering and soft-clustering psPRSs in the meta-analysis of over 18,000 patients from HKDR and HKDB.

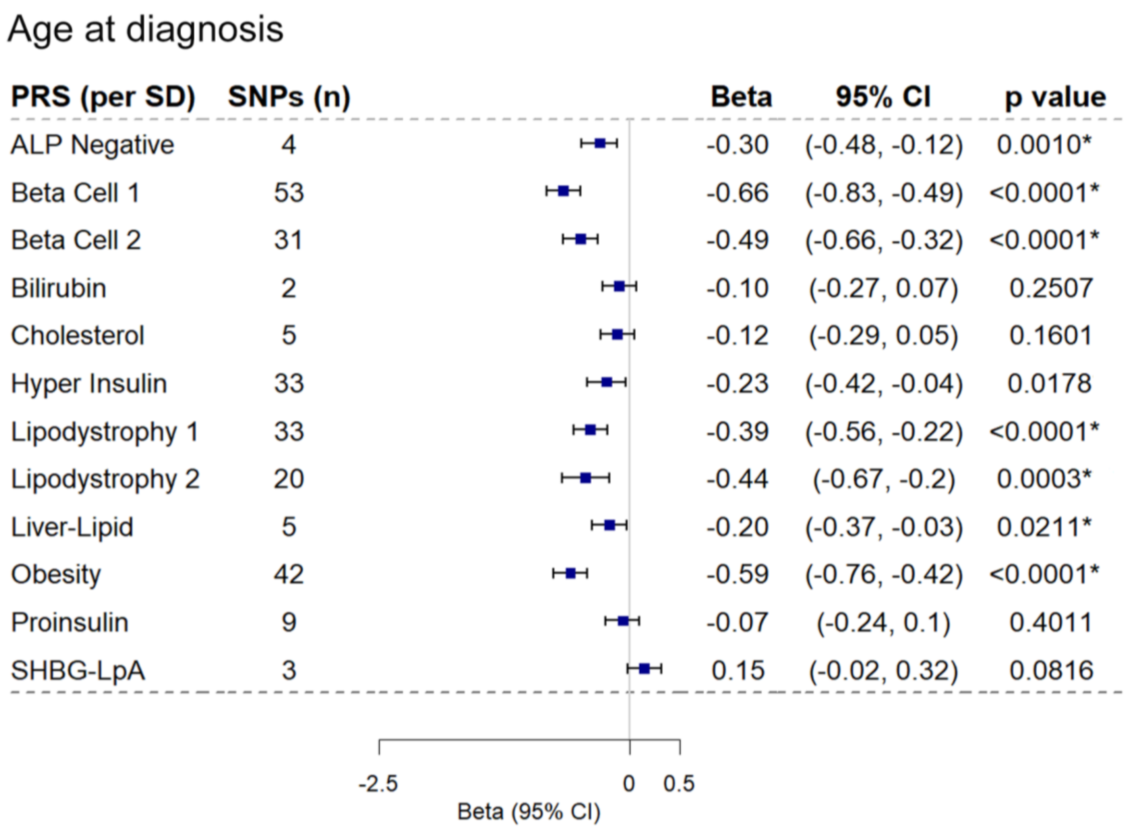

ESM Fig. 2. Associations between soft-clustering psPRSs and age at diagnosis with T2D in the meta-analysis of over 18,000 patients from HKDR and HKDB. Betas were adjusted for sex and top 4 PCs. One asterisk (*) indicates Bonferroni corrected $p < 0.05$.

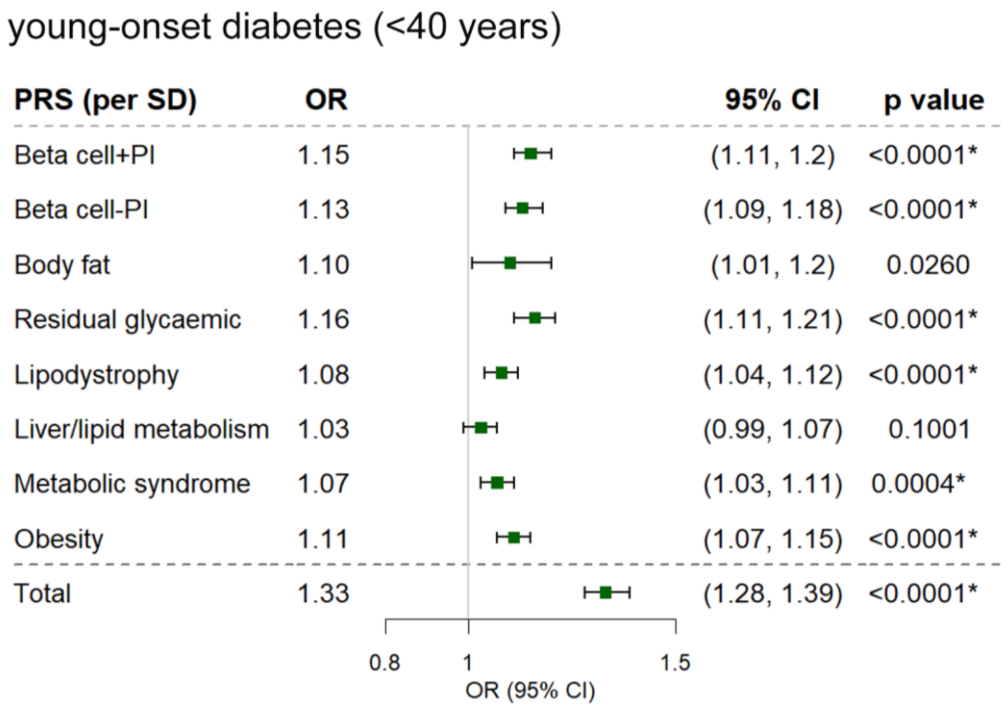

ESM Fig. 3. Associations between hard-clustering psPRSs and total T2D PRS, and young-onset diabetes with T2D in the meta-analysis of over 18,000 patients from HKDR and HKDB. Odds ratio (OR) were adjusted for age, sex, T2D duration and top 4 PCs. One asterisk (*) indicates Bonferroni corrected $p < 0.05$.

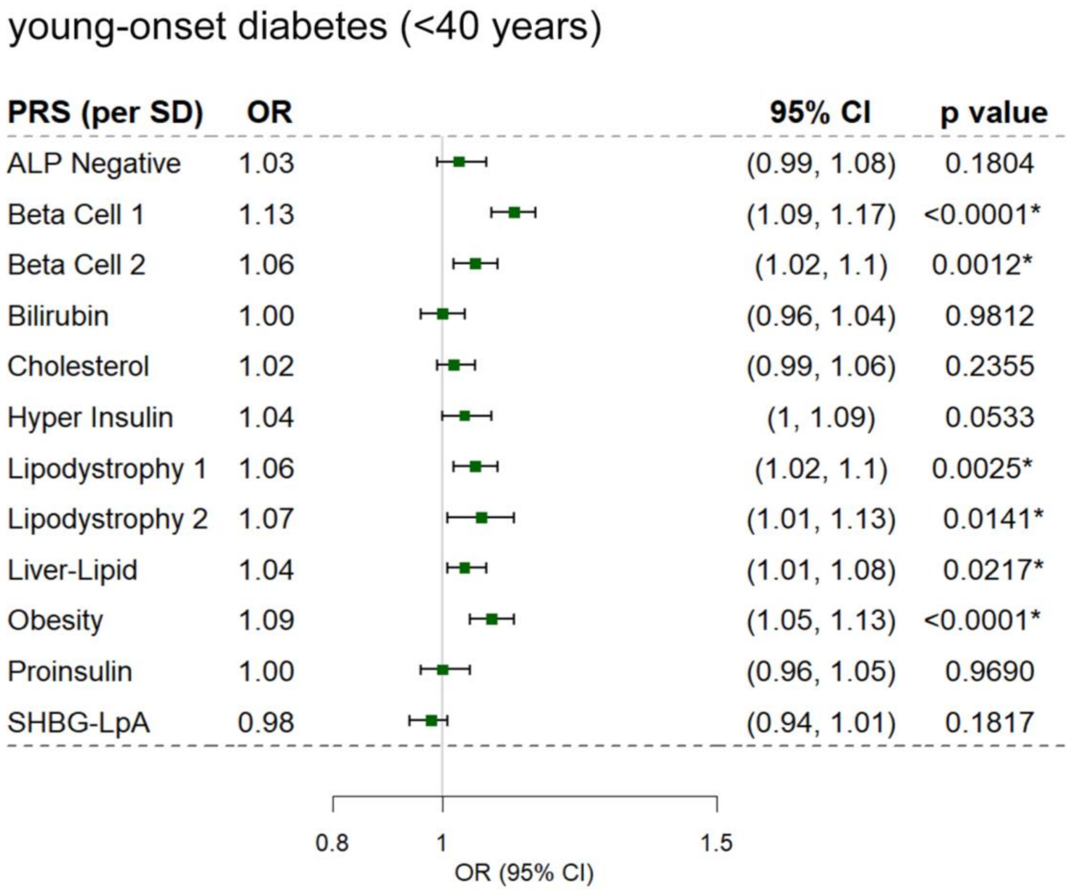

ESM Fig. 4. Associations between soft-clustering psPRSs and young-onset diabetes in the meta-analysis of over 18,000 patients from HKDR and HKDB. Odds ratio (OR) were adjusted for age, sex, T2D duration and top 4 PCs. One asterisk (*) indicates Bonferroni corrected $p < 0.05$.

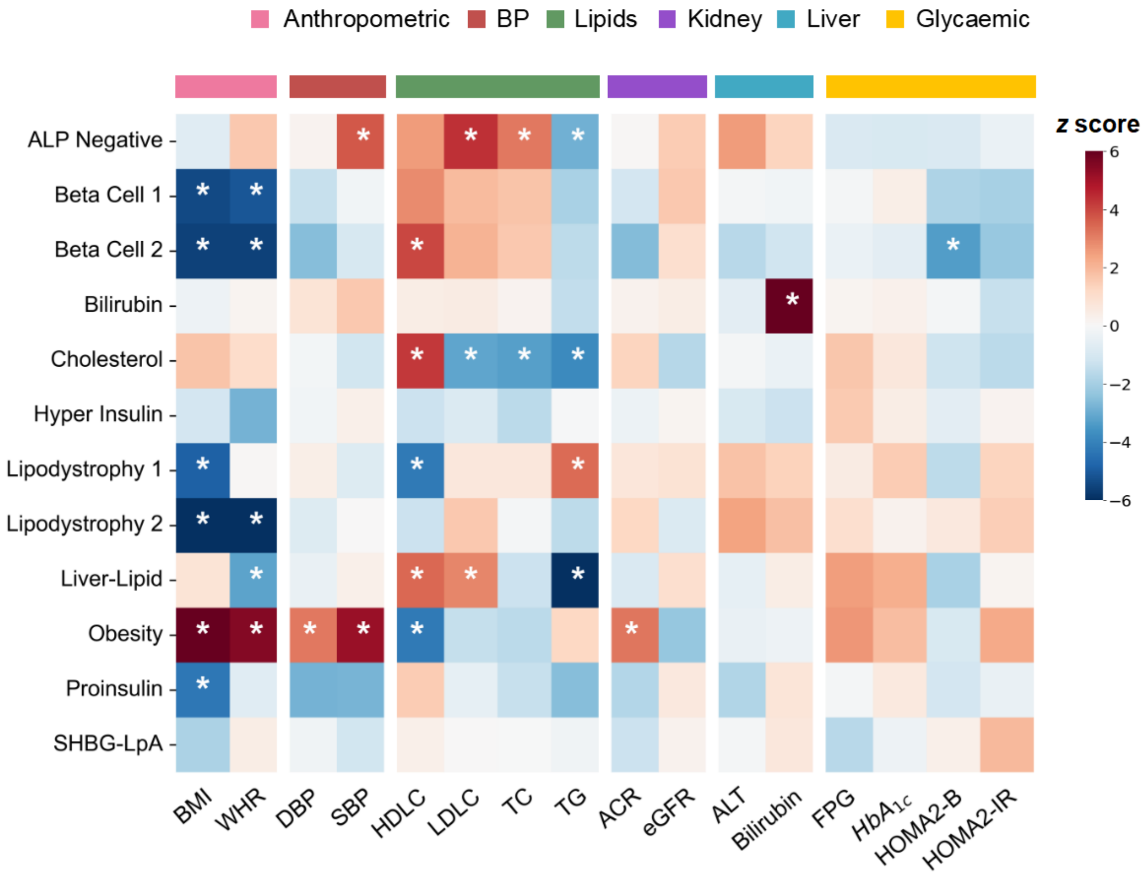

ESM Fig. 5. Associations between soft-clustering psPRSs and baseline clinical variables in the meta-analysis of over 18,000 patients from HKDR and HKDB. Age, sex, duration of T2D and top 4 PCs were adjusted. The colorbar indicates the z-score of the regression coefficient. One asterisk (*) indicates Bonferroni corrected $p < 0.05$.

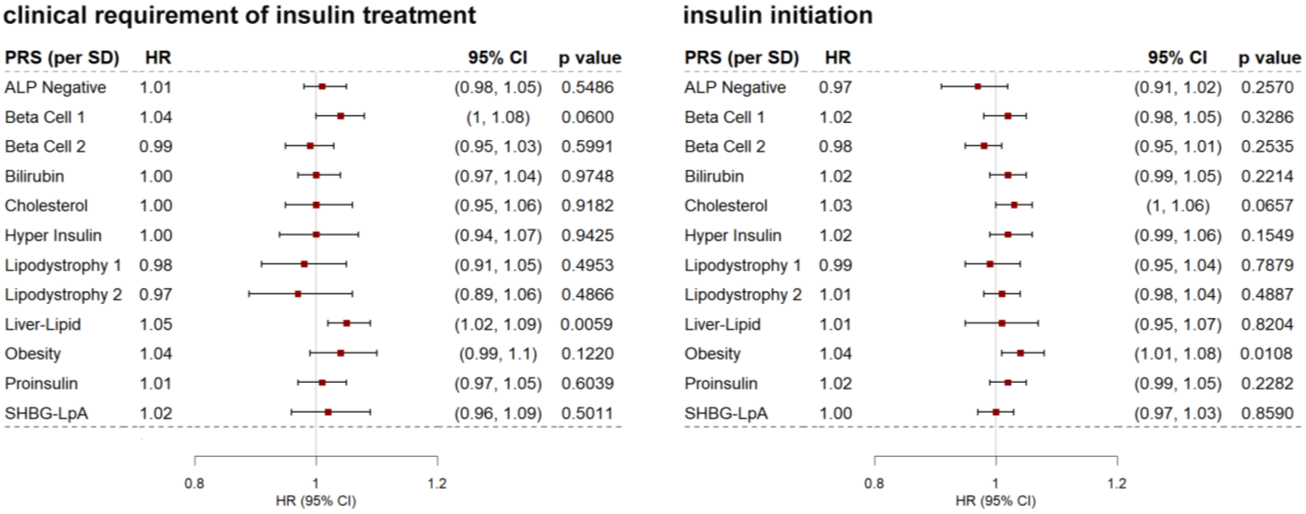

ESM Fig. 6. Prospective associations between soft-clustering psPRSs and clinical requirement of insulin treatment and insulin initiation in HKDR and HKDB. Age, sex, duration of T2D and top 4 PCs were adjusted. One asterisk (*) indicates Bonferroni corrected $p < 0.05$. The error bars indicate 95% CI (confidence interval). For the numbers of progressors and non-progressors of each outcome, please see the ESM Table 1.

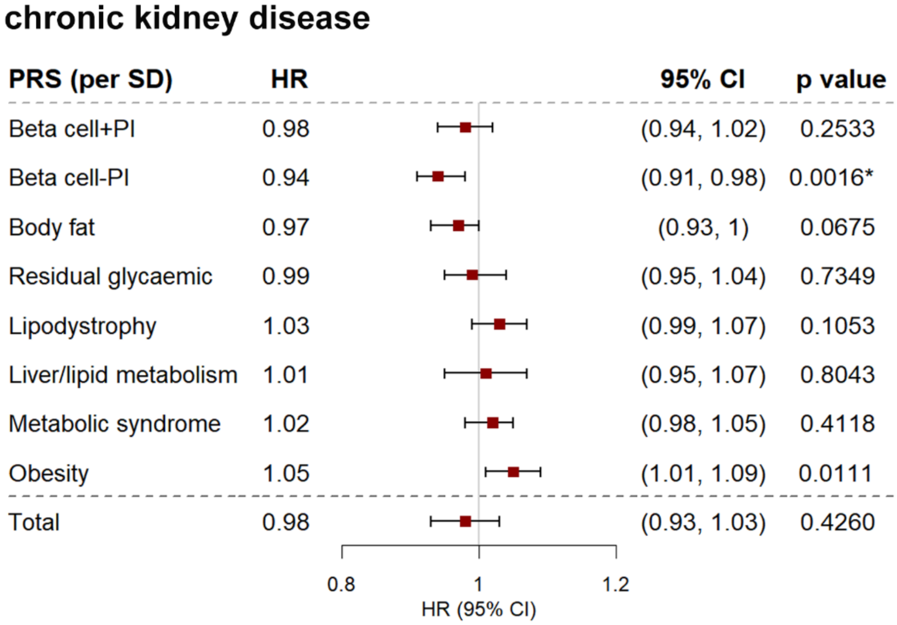

ESM Fig. 7. Prospective associations between hard-clustering psPRSs and total T2D PRS, and chronic kidney disease in HKDR and HKDB. Age, sex, duration of T2D and top 4 PCs were adjusted. One asterisk (*) indicates Bonferroni corrected $p < 0.05$. The error bars indicate 95% CI (confidence interval). For the numbers of progressors and non-progressors of each outcome, please see ESM Table 1.

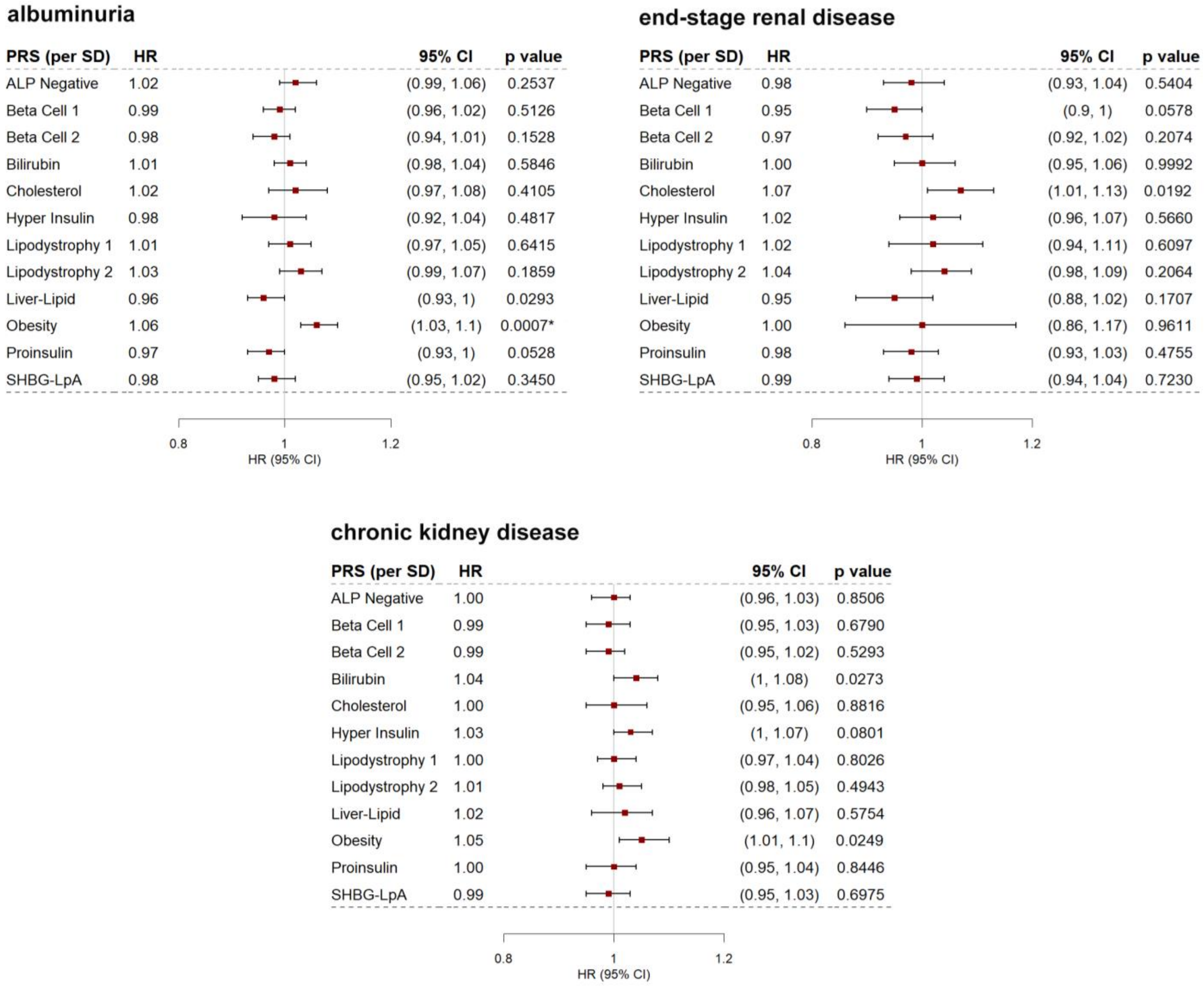

ESM Fig. 8. Prospective associations between soft-clustering psPRSs and albuminuria, end-stage renal disease and chronic kidney disease in HKDR and HKDB. Age, sex, duration of T2D and top 4 PCs were adjusted. One asterisk (*) indicates Bonferroni corrected $p < 0.05$. The error bars indicate 95% CI (confidence interval). For the numbers of progressors and non-progressors of each outcome, please see ESM Table 1.

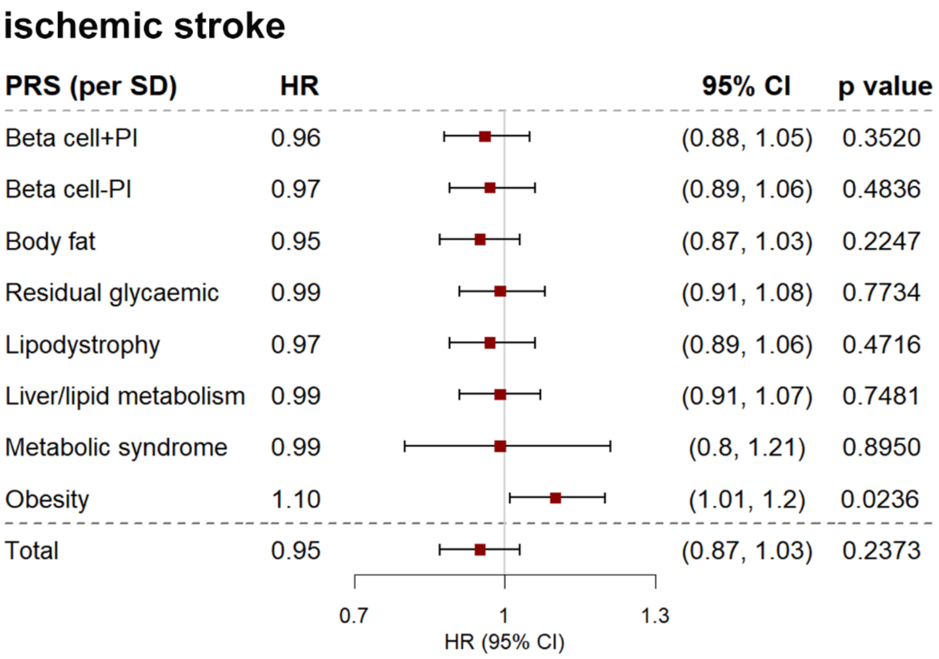

ESM Fig. 9. Prospective associations between hard-clustering psPRSs and total T2D PRS, and ischemic stroke in HKDR and HKDB. Age, sex, duration of T2D and top 4 PCs were adjusted. One asterisk (*) indicates Bonferroni corrected $p < 0.05$. The error bars indicate 95% CI (confidence interval). For the numbers of progressors and non-progressors of each outcome, please see ESM Table 1.

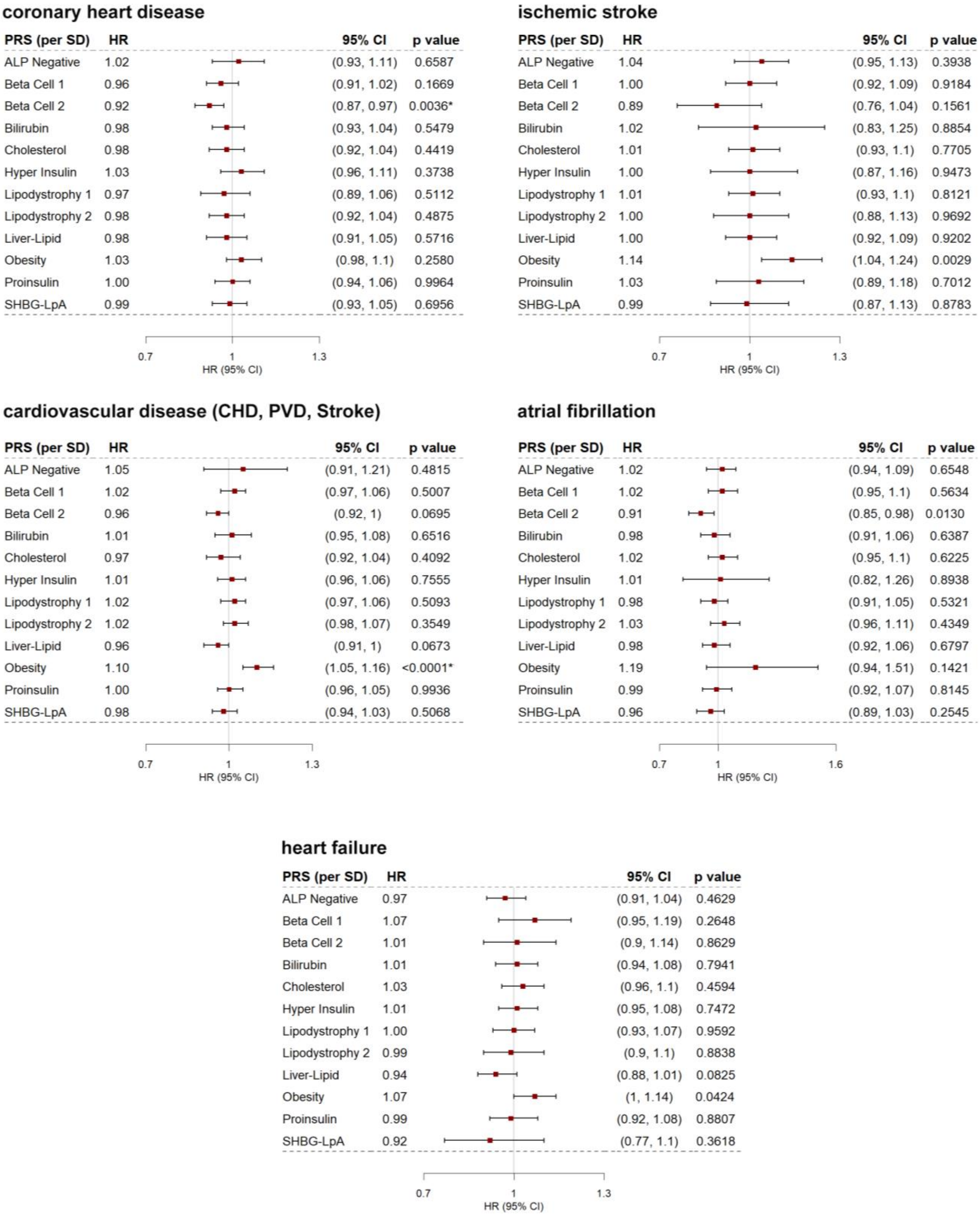

ESM Fig. 10. Prospective associations between soft-clustering psPRSs and incident coronary heart disease, ischemic stroke, cardiovascular disease, atrial fibrillation and heart failure in HKDR and HKDB. Age, sex, duration of T2D and top 4 PCs were

adjusted. One asterisk (*) indicates Bonferroni corrected $p < 0.05$. The error bars indicate 95% CI (confidence interval). For the numbers of progressors and non-progressors of each outcome, please see ESM Table 1.

References

1. Jiang G, Luk AOY, Tam CHT, Xie F, Carstensen B, Lau ESH, Lim CKP, Lee HM, Ng ACW, Ng MCY, Ozaki R, Kong APS, Chow CC, Yang X, Lan HY, Tsui SKW, Fan X, Szeto CC, So WY, Chan JCN, Ma RCW, Hong Kong Diabetes Register TRSSG. Progression of diabetic kidney disease and trajectory of kidney function decline in Chinese patients with Type 2 diabetes. Kidney Int 2019;95:178-187
2. Stevens PE, Ahmed SB, Carrero JJ, Foster B, Francis A, Hall RK, Herrington WG, Hill G, Inker LA, Kazancıoğlu R. KDIGO 2024 clinical practice guideline for the evaluation and management of chronic kidney disease. Kidney international 2024;105:S117-S314
